# Instrumentation for natural orifice translumenal endoscopic surgery and laparoendoscopic single-site surgery

**DOI:** 10.4103/0970-1591.70577

**Published:** 2010

**Authors:** Candace F. Granberg, Matthew T. Gettman

**Affiliations:** Department of Urology, Mayo Clinic, Rochester, MN, USA

**Keywords:** Instruments, laparoendoscopic single-site surgery, natural orifice translumenal endoscopic surgery

## Abstract

**Objective:**

To describe the evolution of instrumentation and technology for natural orifice translumenal endoscopic surgery (NOTES) and laparoendoscopic single-site surgery (LESS) as applied to urologic procedures.

**Materials and Methods:**

We performed a search of published reports on PubMed and MEDLINE for the search terms NOTES, single-port, single-incision, single-site, natural orifice + surgery, SPA, LESS, incisionless, and scarless from 1990-2009. Studies relevant to this urologic symposium were chosen for detailed review.

**Results:**

Multiple case reports, case series, and review articles relevant to NOTES and LESS utilized for urologic surgery dating from 1991 to 2009 were identified. We were subsequently able to chronicle the technological advances in instrumentation utilized for NOTES, including transvaginal nephrectomy, transvesical NOTES, combination or hybrid NOTES, and robotic-assisted NOTES or R-NOTES. For LESS, we detailed the development of various access ports and operating platforms to facilitate performing urologic procedures through a single-port access site.

**Conclusions:**

Significant progress has been made in developing new, multi-lumenal access ports and articulating or curved instruments to aid in triangulation necessary for certain urologic procedures. Magnetic anchoring guidance systems (MAGS) have further enhanced the approach to LESS, with the potential for future application to NOTES. NOTES and LESS have future implications for the armamentarium of urologic surgeons, although much more research is necessary to further improve instrumentation and overcome the learning curve necessary for new technology.

## INTRODUCTION

Over the past two decades, there has been a significant shift in the approach to surgery, with patients and surgeons alike striving for fewer, smaller incisions with the goals of a hastened postoperative convalescence as well as improved cosmesis. To accommodate this shift, technology and instrumentation needed for various minimally-invasive approaches have undergone numerous modifications. In 1991, Clayman *et al*. performed the first successful laparoscopic nephrectomy through multiple keyhole incisions.[1] Since then, techniques have been developed to permit laparoscopic instrumentation through a single incision, or laparoendoscopic single-site surgery (LESS). Additionally, natural orifice translumenal endoscopic surgery (NOTES) eliminates entirely the need for abdominal incisions. Here, we review the evolution of instrumentation and technology for NOTES and LESS as applied to urologic surgery.

## NOTES

### Transvaginal nephrectomy

In 2002, Gettman *et al*.[2] assessed the feasibility of transvaginal laparoscopic nephrectomy in the porcine model. Pneumoperitoneum was established transabdominally with a Veress needle, and modified plastic fascial dilators traditionally used with the Amplatz dilating system (Microvasive, Boston Scientific, Watertown, MA) were used as transvaginal laparoscopic ports. The posterior colpotomy was balloon dilated to 24Fr (UroMax II Plus, Microvasive) over a guidewire. Flexible cystoscope or standard 5-mm laparoscopic lens and camera were utilized transvaginally, and in some cases a traditional 5-mm transabdominal port was placed for the camera. Standard laparoscopic instruments, and in some cases articulating instruments (Roticulator Endo Dissect and Roticulator Endo Mini-Shears, U.S. Surgical, Norwalk, CT) were used for dissection. The Endo-GIA stapler (U.S. Surgical) was used transvaginally to divide hilar vessels and the ureter, and a 10-mm EndoCatch II device (U.S. Surgical) was used to entrap the specimen. Obstacles discovered during this investigation included limitations inherent to standard laparoscopic instruments, as well as anatomic constraints of the porcine model.

Clayman *et al*.[3] expanded on Gettman’s initial experience with transvaginal nephrectomy, using a novel platform in a female farm pig. Again, pneumoperitoneum was obtained transabdominally with a Veress needle. Transvaginally, a TransPort Multi-Lumen Operating Platform (USGI Medical, San Clemente, CA) with 4 working channels (two 4-mm, one 6-mm, one 7-mm) was placed through a posterior colpotomy incised with an endoscopic needle knife (Cook Medical Inc., Bloomington, IN). Tissue-acquisition devices (g-Prox and g-Lix, USGI Medical, San Clemente) were used via the TransPort for retraction in combination with other endoscopic instruments. An additional 12-mm transabdominal port was placed to maintain pneumoperitoneum, confirm placement during transvaginal access, place a fan retractor, and apply a vascular GIA (Ethicon Endo-Surgery, Inc, Cincinnati, OH) for division of the hilar vessels. The specimen was removed transvaginally utilizing a 10-mm EndoPouch (Ethicon Endo-Surgery) placed via the 12-mm transabdominal port. Although the TransPort allowed for multiple instruments to be utilized through a single port, drawbacks with this experiment included difficulty with triangulation and inability to secure the renal hilum through the channels in this platform.

### Transvesical NOTES

Use of the bladder as a portal of entry for NOTES was demonstrated by in eight female pigs by Lima *et al*. in 2006.[4] Under ureteroscopic guidance, the bladder was incised with Olympus A2576 scissors placed through the working channel of the ureteroscope. Next, a 5Fr open-ended ureteral catheter was advanced through the incision into the peritoneal cavity. Over a guidewire placed through the ureteral catheter, the cystotomy was dilated using the dilator of an ureterorenoscope sheath (Microvasive-Boston Scientific Corp., Natick MA) encased by a flexible overtube. The ureteroscope could subsequently be passed through the overtube into the peritoneum, through which an Olympus UHI-3 Insufflator was used to create pneumoperitoneum, and transvesical peritoneoscopy was feasible.

Gettman and Blute[5] subsequently reported the first clinical application of transvesical NOTES in a patient undergoing robotic prostatectomy with established transabdominal pneumoperitoneum. Through a rigid cystoscope placed transurethrally, a flexible injection needle was advanced through the bladder wall, and guide wire was advanced through the needle. Balloon dilation (UroMax, Boston Scientific) was performed to dilate the cystotomy tract, and a flexible cystoscope could then be advanced over the guidewire to perform peritoneoscopy.

### Hybrid NOTES

There have been several reports of investigators using more than one natural orifice to perform NOTES, termed a hybrid approach. In 2006, Lima *et al*.[6] reported successful porcine nephrectomy via a combined transgastric and transvesical approach. A 5-mm camera was first placed transvesically, via their technique outlined previously in this review. Gastrotomy was established on the anterior wall with a needle knife and cautery and enlarged subsequently with a papillotomy knife. By alternating a rigid Olympus A2942A ureteroscope and a double channel Olympus GIF-2T160 endoscope, the working channels of both portals were able to be used for instrumentation. UltraCision Harmonic Scalpel Long Shears ultrasonic scissors were used via the transvesical port for dividing the renal artery and vein. In two animals, the ultrasonic scissors were insufficient to control the vessels, and a Ligamax 5 clip applicator was successfully used to place clips for hemostasis. A major limitation in this study was the inability to close the gastrotomy portal safely. Additionally, instrumentation was limited for extraction, thus specimens were left *in situ*.

Isariyawongse *et al*.[7] built on this concept, performing nephrectomy via a hybrid transgastric and transvaginal approach in a porcine model. Transgastric access was obtained first, with subsequent pneumoperitoneum established through a single-lumen therapeutic gastroscope (Olympus EVIS Type 100 Q140). Under transgastric visual guidance, needle-knife electrocautery was used to create a transvaginal port for a 5.9mm single-channel pediatric gastroscope (Olympus GIF XP-160, Center Valley, PA). A novel laparoscopic trocar/endoscopic overtube was advanced over the transvaginal endoscope as the NOTES port and portal for continued insufflation. Standard laparoscopic instruments were used for dissection, and an Endo GIA Universal 12mm stapler (AutoSuture, Norwalk, CT) was successfully used via the transvaginal trocar to divide the hilar vessels and ureter. The specimen was entrapped in a retrieval device (Endocatch Gold 10mm, AutoSuture, Norwalk, CT) and removed through the vaginal incision.

### Hybrid R-NOTES

The success of robotic-assisted laparoscopic surgery as applied to urologic procedures prompted Box *et al*.[8] to attempt a robotic-assisted hybrid NOTES nephrectomy in a female farm pig. A 12mm transabdominal port was placed midline for use of the robotic camera as well as maintaining pneumoperitoneum. Two 12mm standard laparoscopic ports (Ethicon Endosurgery, Cincinnati, OH) were placed through the vagina and colon, with robotic ports telescoped through these ports upon docking of the robot. Robotic instruments (HotShears and ProGrasp) were used for dissection, and the hilum was divided using a vascular Endo GIA stapler via the transvaginal port. The specimen was withdrawn through the vaginal incision. A significant limitation in this study was difficulty with robotic arm movement due to proximity of the ports, which led to frequent collisions and “sword-fighting”.

Haber *et al*.[9] hoped to overcome the lack of triangulation in previous NOTES procedures necessary for suturing by applying the robot to a hybrid transabdominal single-port with a transvaginal port for dismembered pyeloplasty, partial nephrectomy, and radical nephrectomy in porcine models. The transabdominal port consisted of either a single port with 3 channels (Uni-X, Pnavel Systems, Morganville, NJ) placed in the umbilicus or two ports (12mm standard trocar and 8mm robotic trocar) placed through a single 2cm umbilical incision. Under laparoscopic vision, a flexible 12mm cannula 20cm long (US Endoscopy, Mentor, OH) was placed as a transvaginal port, to which a robotic arm was attached. For pyeloplasty, EndoWrist needle drivers (Intuitive Surgical, Inc., Sunnyvale, CA) were used for the anastomosis. For radical nephrectomy, the renal artery was clipped and divided, and an XL Endo-GIA stapler (Covidien, Mansfield, MA) was used to control the renal vein, with introduction of the clip applier and stapler via the vaginal port. For partial nephrectomy, the vaginal port served as an access point for introducing sutures, bolsters, and a laparoscopic bulldog (MicroFrance, Saint-Aubin le Monial, France) for vascular control and renorrhaphy. Although collisions between the umbilical robotic arm and laparoscope still occurred in a number of the cases due to their parallel alignment, the addition of the robot to NOTES provided a great advantage in providing 3-D vision, articulating instruments, and improved ability for intracorporeal suturing.

## LAPAROENDOSCOPIC SINGLE-SITE SURGERY

To overcome the issue of “sword-fighting” due to ports in close proximity, a single port with multichannel access was developed through which specially designed, curved laparoscopic instruments could be introduced. The Uni-X Single Port Access Laparoscopic System (Pnavel Systems, Morganville, NJ) was used by Kaouk *et al*.[10] to perform renal cryotherapy, wedge kidney biopsy, radical nephrectomy, and abdominal sacrocolopexy. Two of the cryotherapy cases were performed with a retroperitoneoscopic approach, with the multichannel port placed at the tip of the 12^th^ rib using an open Hassan technique. The other cases were performed transperitoneally, with the port placed through a 1.5cm incision at the inner edge of the umbilicus. The 5mm laparoscope used in these cases had a flexible, steerable tip with an incorporated light source within the camera head (Olympus Surgical, Orangeburg, NY). For the retroperitoneoscopic cryotherapy cases, a 100mm flexible, steerable ultrasound probe was advanced alongside the port incision for localization. Nephrectomy did require an additional 10mm port for introduction of a laparoscopic stapler, and through this port site the specimen was extracted after extending the incision. During sacrocolpopexy, mesh was passed perivaginally under direct vision (laparoscopically and vaginally), and secured using a combination of sutures and a 5mm Pro-Tack tacking device (U.S. Surgical). In this series, although the advent of curved instruments theoretically should have decreased the collision of instruments, it still occurred quite commonly. Additionally, the learning curve for this technique for such instruments requires additional training prior to clinical use.

Desai *et al*.[11] reported on the first clinical use of a novel single port device (R-Port, Advanced Surgical Concepts, Dublin, Ireland) placed transumbilically for laparoscopic nephrectomy and dismembered pyeloplasty. This valve component of the device has either three inlets (one for 12mm instrument, two for 5mm instruments) or four inlets (two for 12mm and two for 5mm). A 5mm rigid 30 ° video-laparoscope (EndoEye, Olympus Medical, Tokyo, Japan) was utilized. Curved instruments (Advanced Medical Concepts) were used during the nephrectomy. The 12mm inlet allowed for a 10mm Hem-O-Lok (Teleflex Medical, Research Triangle Park, NC) clip applier to be used for hilar control during nephrectomy, and the specimen was extracted through the umbilical incision. Pyeloplasty required an additional 2mm needlescopic port for placement of a needlescopic grasper for retraction and triangulation. Since their initial report, Desai *et al*. reported on 100 patients who have successfully undergone a variety of procedures using the R-Port device.[12] Additionally, Rane *et al*.[13] and Gill *et al*.[14] have reported on simple nephrectomies and live donor nephrectomy, respectively, utilizing the R-Port with transumbilical specimen extraction.

In pediatric urology, LESS has been used successfully to perform varicocelectomy, nephrectomy, and nephroureterectomy. For LESS varicocelectomy in three adolescents, Kaouk and Palmer[15] utilized the Uni-X Single Port Access Laparoscopic System (Pnavel Systems, NJ) placed at the umbilicus along with a curved laparoscopic scissors and grasper, with a standard clip applier used for vascular control. Park *et al*.[16] used a homemade single-port device through a single umbilical incision to perform LESS nephrectomy in a three-year-old girl. They utilized an extra-small Alexis wound retractor (Applied Medical, Rancho Santa Margarita, CA) placed at the umbilicus with a surgical glove fixed to the retractor. The fingers of the glove were used as trocar sites for a 12mm trocar and two 5mm trocars. Dissection was carried out with standard laparoscopic instruments and a 5mm flexible laparoscope with integrated camera (EndoEye, Olympus, Orangeburg, NY), and the specimen extracted through the umbilical incision. To perform LESS nephroureterectomy in a 10-year-old girl, Bayazit *et al*.[17] used three 5mm small head trocars (LiNA Medical, Glostrup, Denmark) through a 2cm umbilical incision, as a SPA-specific access device was not available. They were able to successfully perform nephroureterectomy using standard laparoscopic instruments, however they noted “sword-fighting” of the instruments and the laparoscope, and triangulation with this technique was difficult.

To further improve the working space of LESS, magnetic anchoring guidance systems (MAGS) were developed to eliminate the camera as a space-occupying port. With this system, a magnet- or needle-lockable platform is placed within the abdomen, is stabilized by an external magnetic element on the anterior abdominal wall, and can be moved within the abdomen. Zeltser and Caddedu[18] described the use of this system to perform single-trocar laparoscopic porcine nephrectomy. The MAGS internal component was inserted through a single 12mm transumbilical trocar. A “light“ trocar was placed through the same incision, which was also used for passing grasping forceps and a laparoscopic stapler, and successful nephrectomy was performed.

Caddedu *et al*.[19] further expounded on this technology by applying MAGS in the clinical setting. Nephrectomy was performed utilizing a 2.5cm umbilical incision, deploying the MAGS camera, and placing a four-channel single-access port (QuadPort, QuSurgical Concepts, Dublin, Ireland). After positioning the MAGS camera utilizing direct vision employed by a standard laparoscope, the remainder of the case was performed exclusively using the MAGS camera and conventional laparoscopic instruments. The kidney and MAGS camera were removed via the umbilical incision.

Advantages of the MAGS system include improved triangulation and visualization during LESS. The camera can be easily manipulated around the abdomen when necessary to facilitate visualization. Moreover, the camera is not a source of collision among working instruments within a single incision. Drawbacks to this system include decreasing coupling strength of the magnets, restriction of use to non-obese patients, and need for an exit site for the wires required for power and image transmission.

## CONCLUSION

Significant advancements have been made in development of technology and instrumentation that can be deployed through either natural orifices or single ports to perform urologic procedures. Although there have been several encouraging reports of successful urologic surgery performed via NOTES and LESS, further research is necessary to develop improved articulating instruments, miniaturized instruments, and operating platforms to enhance range-of-motion, visualization, and triangulation. Future urologists can look forward to having NOTES and LESS in their armamentarium, understanding that in addition to the need for continued improvement of instrumentation, there is a learning curve that will need to be overcome.
